# Meetings of Societies

**Published:** 1914-06

**Authors:** 


### MEETINGS OF SOCIETIES.

### Bristol Medico-Chirurgical Society.

*March 11th*, 1914.

Dr. P. Watson-Williams, President, in the Chair.

Mr. Hey Groves exhibited a loan collection of **Photographs, skiagraphs, and instruments,** from Germany, Austria, France, Belgium, America, and the United Kingdom, illustrating the operative treatment of fractures.

Dr. Edgeworth read notes of a case of **Uniformly enlarged thyroid with tachycardia, diagnosed and treated for six years as an incomplete form of exophthalmic goitre, and cured by administration of thyroid extract** (see p. 144).—Dr. J. O. Symes asked if the patient lost weight under thyroid treatment. A possible explanation of the recovery was that it took place spontaneously and independently of the treatment. A great deal of thyroid extract, etc., on the market was inert, and therefore not harmful.—The President asked if there was any other evidence of pressure on the vagus, and alluded to cases of mixed hyperthyroidism and myxœdema.—Dr. Edgeworth replied that the patient had not a single symptom of myxædema. The pulse rate of 100 was against it. Improvement began a week after taking the thyroid extract. He did not believe a large number of cases recovered spontaneously. Only 25 per cent. of cases get well in that way, according to Mackenzie and Hale White. The thyroid gland in his case had become much smaller under the treatment.

Mr. C. F. Walters read a paper on **Autoplastic intra-medullary bone pegging of fractures** (see p. 139).

Mr. Hey Groves discussed **Some modern ideas in the treatment of fractures.**—Mr. Greer (Newport) alluded to the need for rapidity in operating. He considered Lambotte's methods superior to Lane's.—Mr. Sheen (Cardiff) pointed out that though a skiagram showed a good anatomical result, it did not reveal the suppuration nor the stiffness which might follow an operation. On the other hand, the result of treatment might appear bad in a skiagram, but the functional capacity of the limb be good. We ought to compare a series of patients who had been treated by operation with a series treated without operation.—Mr. Walters and Mr. Groves replied.

*April 8th,* 1914.

Dr. P. WATSON-WILLIAMS, President, in the Chair.

The following cases were shown :—

Dr. MICHELL CLARKE : (*a*) **A case of goitre, with symptoms of myxœdema, which had been relieved by the administration of a thyroid extract.** — Mr. HEY GROVES advised removal of a portion of the gland to relieve pressure symptoms, and considered that the presence of myxœdema did not contra indicate this, as the gland was not functioning normally. (*b*) **A case of acute toxic polyneuritis, which simulated an acute ascending spinal palsy in its origin.** No cause for it could be ascertained, but the condition was improving. (*c*) **A case of septic rheumatic endocarditis, of fifteen months' duration, in a man aged 19.** Pyrexia and joint swellings occurred whenever the patient got up from bed. The pyrexia and rheumatism ceased after administration of normal sterile horse serum by the rectum.

Dr. J. A. NIXON : (*a*) **A case of multiple or "button" epitheliomata of the leg, which appeared subsequent to the treatment of an extensive lupus with X-rays.** The case, it was thought, suggested that the frequent administration of small doses of X-rays was the most likely method to lead to this result. (*b*) **A case of progressive atrophy of the right hand and forearm of an adult woman.** It was associated with what seemed to be a congenital malformation of both elbow-joints.— Mr. HEY GROVES suggested that the atrophy might be due to a nerve lesion, the result of a bone malformation. (*c*) **A case for diagnosis resembling Friedreich's ataxia.**

Mr. HEY GROVES : **Two patients in whom autoplastic bone grafts had been used with success.** In one case a portion of the ulna had been removed and wired on to form a splint for an ununited fracture of that bone, and in the other a peg from the tibia had been used to fix a movable knee-joint after an excision. The exhibitor insisted on the necessity of firm mechanical fixation of a graft to obtain union.

Mr. A. J. WRIGHT : **A case of necrosis of the superior maxilla and mandible in a patient with extensive laryngeal and pulmonary tuberculosis.** The condition was progressive, and had not been influenced by the administration of salvarsan, mercury, and iodides.

Dr. E. CECIL WILLIAMS read notes on a **Case of acute lymphatic leukæmia** (see p. 147).

*May 13th*, 1914.

Dr. P. WATSON-WILLIAMS, President, in the Chair.

The meeting was devoted to a discussion on **The results of the treatment of pulmonary and laryngeal tuberculosis by vaccines** (see p. 97).—The discussion was opened by Dr. S. H. HABERSHON, Physician to Brompton Hospital, who dealt with "The secondary infections in cases of pulmonary tuberculosis, with special reference to their vaccine treatment."—Dr. NOEL BARDSWELL, Medical Superintendent of the King Edward Sanatorium, followed, and discussed "Tuberculin treatment."—Drs. SHINGLETON SMITH, L. J. SHORT, NEWMAN NEILD and NIXON took part in the discussion.

J. LACY FIRTH,
A. J. WRIGHT, *Hon. Sec.*

---

